# A Strong Decrease in *TIMP3* Expression Mediated by the Presence of *miR-17* and *20a* Enables Extracellular Matrix Remodeling in the NSCLC Lesion Surroundings

**DOI:** 10.3389/fonc.2019.01372

**Published:** 2019-12-13

**Authors:** Karolina H. Czarnecka, Bartosz Szmyd, Magda Barańska, Marcin Kaszkowiak, Jacek Kordiak, Adam Antczak, Dorota Pastuszak-Lewandoska, Ewa Brzeziańska-Lasota

**Affiliations:** ^1^Department of Biomedicine and Genetics, Medical University of Lodz, Łódz, Poland; ^2^Quantitative Genomic Medicine Laboratories, S.L., Esplugues de Llobregat, Barcelona, Spain; ^3^Department of Chest Surgery, General and Oncological Surgery, University Teaching Hospital No. 2, Medical University of Lodz, Łódz, Poland; ^4^Department of General and Oncological Pulmonology, Medical University of Lodz, Łódz, Poland; ^5^Department of Microbiology and Laboratory Medical Immunology, Medical University of Lodz, Łódz, Poland

**Keywords:** NSCLC molecular diagnostic markers, miRNA regulation, microRNA, extracellular matrix remodeling, metalloproteinases, tissue inhibitors of metalloproteinases, exosomes

## Abstract

**Background:** Lung cancer is one of the most common causes of death worldwide with a relatively high fatality rate and a mean 5-years survival of about 18%. One of the hallmarks of cancer is the extracellular matrix (ECM) remodeling, which is crucial for metastasis. This process may be regulated by miRs targeting metalloproteinases (MMPs) associated with the ECM breakdown and metastatic process or blocking the action of tissue inhibitors of metalloproteinases (TIMPs). Search for early biomarkers is essential in detecting non-small cell lung cancer (NSCLC) and distinguishing its subtypes: Adenocarcinoma (AC) from Squamous Cell Carcinoma (SCC), enabling targeted chemotherapy.

**Methods:** *MiR-17* and *miR-20a* targeting *MMP2* and *TIMP3* were selected by TCGA data analysis with further validation using miRTarBase and literature. The study group comprised 47 patients with primary NSCLC (AC and SCC subtypes). RNA was isolated from the tumor and normal-looking neighboring tissue (NLNT) free of cancer cells. MiRs from peripheral blood exosomes were extracted on admission and 5–7 days after surgery. Gene and miRs expression were assessed in qPCR using TaqMan probes.

**Results:** The *MMP2* has been expressed on a similar level in NLNT, as in cancer. While, *TIMP3* expression was decreased both in cancer tissue and NLNT, with significantly lower expression in cancer. *TIMP3* downregulation in NLNT and in SCC subtype correlated negatively with *miR-20a*. The preoperative *miR-17* expression was significantly higher among patients with SCC compared to AC. Receiver operating characteristic (ROC) analysis of *miR-17* as AC subtype classifier revealed 90% specificity and 48% sensitivity in optimal cut-off point with area under ROC curve (AUC): 0.71 (95%CI: 0.55–0.87). Within NSCLC subtypes: a strong negative correlation between pack-years (PY) and *TIMP3* expression was observed for NLNT in the SCC group.

**Conclusion:** The *TIMP3* silencing observed in the NLNT and its negative correlation with presurgical expression of *miR-20a* (from serum exosomes), suggest that miRs can influence ECM remodeling at a distance from the center of the lesion. The *miRs* expression pattern in serum obtained before surgery significantly differs between AC and SCC subtypes. Moreover, decreased *TIMP3* expression in NLNT (in SCC group) negatively correlates with the amount of tobacco smoked in a lifetime in PY.

## Introduction

Lung cancer (LC) is one of the most prevalent cancers, with 2 million new cases in 2018 and with a relatively high fatality rate: the overall ratio of mortality to incidence is 0.87 (1, 2). It is also one of the leading causes of cancer mortality in most developed countries, representing almost 20% of deaths due to cancer (2). Two main clinical types are distinguished: small cell lung cancer (SCLC) and non-small cell lung cancer (NSCLC). NSCLC accounts for almost 80% of LC, and comprised squamous cell carcinoma (SSC; 30–32% of LC), adenocarcinoma (AC; 10–35%), and large cell cancer (LCC; 10%) (3–5). Five-years survival rates drastically decrease with the cancer stage, from 83% for AJCC stage I (American Joint Committee on Cancer Staging), to ~6.6% for detection at an advanced stage (AJCC stage III/IV) (6, 7). The molecular markers are needed to identify NSCLC at its early stages, predict cancer development, and treatment response. Potential biomarkers may be found among molecules responsible for extracellular matrix (ECM) remodeling: a process driven by matrix metalloproteinases (MMPs) countered by the endogenous tissue inhibitors of metalloproteinases (TIMPs) (8, 9).

MMPs are the proteolytic enzymes degrading the components of the basement membrane, acting in many physiological processes (embryogenesis, angiogenesis, apoptosis, wound healing) and in cancer development (8, 10). Elevated *MMP2* expression was observed in stromal fibroblasts, preneoplastic bronchial squamous lesions and pulmonary carcinoma (both in highly invasive and moderate growth areas) (11–13). In NSCLC, the *MMP2* upregulation has been associated with greater tumor size or distant metastasis (14, 15). The MMPs' action can be specifically inhibited by non-covalent binding of TIMPs, which leads to tumor growth suppression and apoptosis promotion (9, 16, 17). Decreased *TIMP3* expression has been observed in many human cancers, i.e., LC, gastric, hepatic, prostate, and endometrial cancer (18–20).

*MMP2* and *TIMP3* expression is regulated through microRNAs (miRs), in a post-transcriptional epigenetic mechanism, leading to mRNA degradation, or translation inhibition (21, 22). MiRs are considered as promising molecular markers for the non-invasive early diagnosis of NSCLC (18, 21) and can be assessed in an inexpensive and patients-friendly way in the peripheral blood exosomes (23). Up to date, miRs have been described as potential biomarkers detecting early stages of NSCLC (*miR-182, miR-183, miR-210*, and *miR-126*) (24) or distinguishing SCC from AC (*miR-26a;* small *miR* panel—*205-5p, 944)* (15, 25). In our study, we focused on the *miR-17* and *miR-20a* targeting *MMP2* and *TIMP3*; these were selected based on TCGA data with further miRTarBase and literature validation (see section Selection of microRNA Molecules). *MiR-17* and *miR-20a* have a significant impact on the development of cancer throughout the body (26–28). Both miRs share the ability to stimulate cell proliferation and inhibit apoptosis (29). One of the well-characterized actions of *miR-17* is its ability to target *MMP2* and *TIMP3* genes. Elevated *miR-17* expression was correlated with a worse outcome (negative correlation with overall survival and disease-free survival) in hepatocellular and pancreatic cancers (30). *MiR-20* possesses tumor suppressor activity by blocking VEGF-induced endothelial cell migration (31). Moreover, decreased *miR-20a* expression was found to be associated with faster tumor growth and poor prognosis (32).

The present study evaluates the relative expression of selected genes (*MMP2, TIMP3*) and miRs (*miR-17, miR-20a*) engaged in ECM remodeling in histopathologically-confirmed NSCLC. Many studies showed that the elevated concentration of the exosomes detected in cancer patient's serum originated from the cancer (26–28, 33). The idea of our study was to look for preoperative circulating miR, that can be obtained in a patient-friendly way from the peripheral blood, as simple preoperative clinical biomarker, distinguishing histopathological subtypes of NSCLC, as well as pTNM, and AJCC stages.

## Materials and Methods

### Patient Clinical Features and Lung Tissue Samples

The study material comprised lung tissue and serum samples obtained from 47 patients diagnosed with NSCLC admitted to the Department of Thoracic Surgery, General and Oncologic Surgery (University Teaching Hospital No. 2 in Lodz., Medical University of Lodz, Poland) between July 2014–March 2017. All patients underwent surgery, based on the results of preoperative assessment. The exclusion criteria included a history of other malignancies, active infectious disease and chemo-, or radiotherapy before the surgery. The study was performed in accordance with the Helsinki Declaration and was approved by the Ethical Committee of the Medical University of Lodz, no. RNN/140/10/KE. All participants provided written, informed consent to take part in the study. Detailed characterization of patients, postoperative histopathological verifications of NSCLC samples (according to the WHO Histological Typing of Lung Tumors and IASCLC Staging Project 7th ed.) and patient smoking status estimated in pack-years (PYs) are presented in Table 1.

**Table 1:** Demographic characteristics of patients and histopathological verifications of NSCLC samples.

**Clinical and pathological features**			**Current study**
Entire group			47
Gender		Woman	18
		Men	29
Age group		≤60	10
		>60	37
Histopathological type		AC	24
		SCC	22
pTNM	Tumor size	pT1	12
		pT2	27
		pT3	6
		pT4	1
	Lymph nodes invasion	N0	33
		N1 and N2	13
AJCC		AJCC I	23
		AJCC II	17
		AJCC III	6
Pack-years (PYs)		≤30 PYs	15
		31–45 PYs	14
		>45 PYs	17
Type of the surgery		Lobectomy	33
		Pneumonectomy	9
		Bilobectomy	4
		Segmentectomy	1

*pTNM, International System of Clinico-Morphological Classification of Tumors (TNM, Tumor Node Metastasis) according to the WHO Histological Typing of Lung Tumors; AJCC, American Joint Committee on Cancer Staging (AJCC staging) according to the IASCLC Staging Project 7th edn*.

The NSCLC tumor lung tissue samples were taken from the center of the lesion, while samples of normal-looking neighboring tissue (NLNT) were taken from the surgical margin: histopathological examination confirmed them to be free of cancer cells. After resection, tissue fragments were stored in RNAlater™ Stabilization Solution (Ambion, USA) at −80°C. The peripheral blood samples (~5 ml) were obtained on admission and 5–7 days after surgical treatment. Serum separated by centrifugation was stored at −80°C until exosome isolation.

### Selection of microRNA Molecules

MicroRNAs targeting *MMP2* and/or *TIMP3* in LC were chosen based on the TCGA datasets, containing RNAseq results of NSCLC patients with AC (LUAD project) and SCC (LUSC project) (34–36). Two datasets for AC and SCC, each containing cancer group and a control group, were downloaded using the TCGA biolinks R package. The datasets sizes are presented in Table 2. Further validation, using data retrieved from public microRNA databases (microRNA.org; mirtarbase.mbc.nctu.edu.tw), indicated that *miR-20a* silences *MMP2* expression and *miR-17* targets both *MMP2* and *TIMP3* (see Supplementary Figure 1). In the performed literature search (PubMed query on miR & ECM remodeling & cancer) many studies indicated that both miRs have a significant impact on the development of cancer throughout the body (26–32).

**Table 2:** The size of the obtained dataset from the GDC database.

**Sample type**	**LUAD project**		**LUSC project**	
	**Genes: *MMP2, TIMP3***	**miRs: *miR-17, miR-20a***	**Genes: *MMP2, TIMP3***	**miRs: *miR-17, miR-20a***
Cancer^a^	539	519	502	478
Controls^b^	59	46	51	45

LUAD project, TCGA dataset, containing RNAseq results of NSCLC patients with AC; LUSC project, TCGA dataset, containing RNAseq results of NSCLC patients with SCC;

a “Solid state Tumor” origin selected for cancer samples;

b *“Solid Tissue Normal” origin selected for controls*.

### Gene and miR Expression in the Studied NSCLC Cohort—Laboratory Procedures

#### miR and RNA Isolation and Reverse Transcription

Exosomes were pre-isolated from serum using a standardized isolation kit enabling enrichment of intact exosomes (Total Exosome Isolation Reagent, Invitrogen, USA) and resuspended in PBS. The Total Exosome RNA and Protein Isolation Kit (Invitrogen, USA), standardized for the isolation of 30–120 nm diameter vesicles, was used to recover total RNA including the small RNA fraction (37, 38). The total RNA from tissues was isolated using the Qiagen RNeasy Mini Kit (QIAGEN, USA), according to the manufacturer's protocol. The quality and quantity of RNA was spectrophotometrically assessed. Only samples fulfilling the following requirements were selected for further use: miR concentration 1–5 ng/μl and RNA concentration over 50 ng/μl; with 260/280 nm ratio 1.8–2.0. Reverse transcription (RT) reactions were performed using a High-Capacity cDNA Reverse Transcription Kit with MultiScribe™ Reverse Transcriptase and additional RNase Inhibitor (both Applied Biosystem, USA) according to the manufacturer's protocol.

#### Real-Time Quantitative Polymerase Chain Reactions (Real-Time qPCR)

Real-time qPCRs were performed using the 7900HT Fast Real-Time PCR System (Applied Biosystems, USA). The qPCR mixes for miRs analysis contained: diluted RT product, TaqMan® Universal Master Mix II, without UNG and 1 μl of miR probes: hsa-miR-17-5p or hsa-mir-20a (Applied Biosystems, USA). The miRs expression analysis, performed in DataAssist v3.01, was based on the global normalization method. The qPCR mixes for gene analysis contained diluted cDNA, KAPA PROBE FAST qPCR kit (Kapa Biosystems, USA) and TaqMan assays for *TIMP3* (Gene ID: 7078), *MMP2* (Gene ID: 4313), and *ACTB* (β*-actin*, Gene ID:60) as endogenous control. All reactions were run in triplicate. RNA isolated from normal lung tissue (Human Lung Total RNA, Ambion, USA) served as a calibrator, for which the RQ (relative quantification) value was considered equal to 1. Analysis was based on the comparative ΔΔCT method, according to the formula ΔΔCT = ΔCT test sample—ΔCT calibrator sample.

### Statistical Analysis

Statistical analysis was performed using the Statistica 13.1 (StatSoft, Tulsa, USA). Non-parametric tests were used for statistical analyses, as miR and mRNA expression values did not follow a normal distribution (Shapiro-Wilk test). The results of relative expression analysis (RQ values) are presented as mean ± SD for normal distribution and median value (MV) with interquartile range (IQR) in other cases. When comparing independent groups, ANOVA Kruskal-Wallis, Mann-Whitney *U*-tests (UMW) and the Spearman's rank correlation (r_s_) were used. Expression analysis regarding the pTNM classification was performed according to the tumor size (pT) and presence of node involvement (N). With only one patient in the pT4 group, pT4 was not included in statistical analysis. For the NSCLC subtype determination test, the receiver operating characteristic (ROC) chart was used and to compare the accuracy of various classifiers the area under the ROC curve (AUC) was calculated. TCGA data were analyzed using R software and R/Bioconductor package. *P* < 0.05 was considered as statistically significant.

## Results

### Selection of microRNA Molecules With Genomic Data Commons and miRTarbase

The correlation analysis of miR and gene expression performed on LUAD and LUSC projects data (34–36) revealed statistically significant negative correlations between the examined genes: *MMP2* and *TIMP3* and both miRs (Supplementary Figure 1; Table 3A). Moreover, significant differences were found in miR expression between the cancer and control groups in both NSCLC subtypes. In the LUSC project (SCC), a significant increase of *miR-17* with *miR-20a* decrease of (3.64 times lower) was observed in the cancer group compared to controls (both *p* < 0.001, UMW test). The opposite was found for the AC subtype: a significant decrease of *miR-17* and an increase of *miR-20a* in cancer samples (2.29 times higher) compared to controls (Table 3B).

**Table 3:** Analysis of gene and miR expression in LUAD and LUSC projects: **(A)** Correlation of analyzed miR and gene expression in both projects; **(B)** Gene and miR expression levels in cancer and control groups in both projects.

**(A)** Correlation of analyzed miR and gene expression in LUAD and LUSC projects				
**Squamous cell carcinoma—LUSC**				
		*miR-17*	*miR-20a*	
*MMP2*		−0.47^*^	−0.40^*^	
*TIMP3*		−0.37^*^	−0.34^*^	
**Adenocarcinoma—LUAD**				
		*miR-17*	*miR-20a*	
*MMP2*		−0.33^*^	−0.24^*^	
*TIMP3*		−0.30^*^	−0.25^*^	
**(B)** Analysis of gene and miR expression in cancer and control groups for LUAD and LUSC projects				
	**Gene/miR**	**No of transcripts cancer group [FPKM]**	**No of transcripts control group [FPKM]**	***P*****-value**
LUSC	*MMP2*	6530.92	7945.22	0.095
	*TIMP3*	4780.33	33445.33	**<0.001**
	*miR-17*	958.16	717.86	**<0.001**
	*miR-20a*	79.61	288.56	**<0.001**
LUAD	*MMP2*	5966.76	4919.22	0.388
	*TIMP3*	7539.80	38223.79	**<0.001**
	*miR-17*	626.16	367.34	**<0.001**
	*miR-20a*	195.48	85.93	**<0.001**

* *p < 0.001—in R coefficient analysis*.

*The values marked in bold represent the statistically significant correlations*.

### Genes Expression in Tumor and Normal-Looking Neighboring Tissues

**Figure 1:**
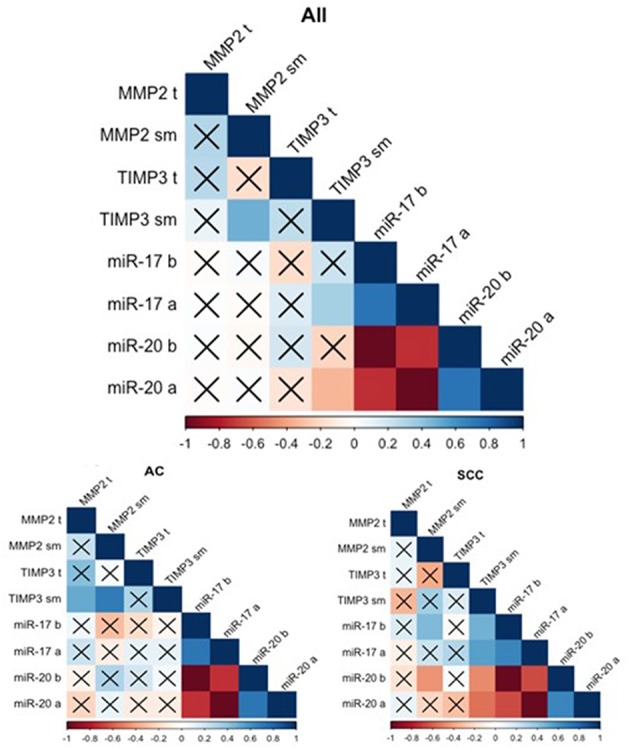
Spearman's rank correlogram of dependencies of miR and gene expression. The lower part represents the dependencies of miR and gene expression in histotypes: Adenocarcinoma (AC) and Squamous Cell Carcinoma (SCC). Correlations marked with X are not statistically significant (*p* > 0.05). Color scale represents Spearman's correlation coefficient (r_s_). t, tumor sample; sm, normal-looking neighboring tissue from surgical margin; b (before), preoperative expression level; a (after), postoperative expression level.

The gene expression analysis for *MMP2* and *TIMP3* was performed on 43 pairs of primary NSCLC tissue and corresponding normal-looking neighboring tissue (see Table 4). In comparison to calibrator (RNA isolated from normal lung tissue), *TIMP3* expression was downregulated in both tissues, and *MMP2* was upregulated in both tissues. The relative distribution and symmetry of gene expression within the analyzed groups (cancer tissue vs. NLNT; AC vs. SCC subtype) are presented in section Materials and Methods in Supplementary Materials and Supplementary Figure 2. The *TIMP3* expression was significantly decreased in cancer tissue in comparison to NLNT (*p* = 0.01; Wilcoxon test; see Supplementary Figure 4). The *MMP2* was expressed on a comparable level in cancer and NLNT (*p* = 0.372; Wilcoxon test). No significant differences were found for gene expression according to age, gender, cancer subtypes, TNM staging (pT and N groups), or AJCC classification. Considering long-life tobacco intake (measured in pack-years) among SCC patients, a negative correlation with *TIMP3* expression in NLNT was found (*R* = −0.68, *p* < 0.001; r_s_). Furthermore, the positive correlation between *TIMP3* and *MMP2* expression was observed in NLNT from surgical margin (*R* = 0.482; *p* = 0.001; r_s_; see Figure 1). In the AC subtype, there were also observed the positive correlations between *TIMP3* in NLNT and *MMP2* levels, both in cancer and NLNT (*R* = 0.699, *p* < 0.001, and *R* = 0.500, *p* = 0.021, respectively; r_s_).

**Table 4:** Clinical and pathological features: median expression level (RQ value) of tested genes.

**Clinical and pathological features**			***N***	***MMP2* NLNT**	***MMP2* cancer tissue**	***TIMP3* NLNT**	***TIMP3* cancer tissue**
Entire group			43	1.422 (IQR: 0.600–3.162)	0.900 (IQR: 0.387–2.392)	0.013 (IQR: 0.006–0.057)	0.006 (IQR: 0.002–0.069)
Gender		Women	14	1.287	0.760	0.021	0.009
		Men	29	1.561	1.213	0.013	0.005
Age group		≤60 years	9	1.330	1.748	0.023	0.010
		>60 years	34	1.446	0.830	0.013	0.005
Histopathological type		AC	21	1.575	1.252	0.013	0.006
		SCC	21	1.015	0.530	0.013	0.004
pTNM	Tumor size	pT1	11	1.471	0.900	0.013	0.004
		pT2	24	1.073	0.479	0.012	0.012
		pT3	6	2.752	1.414	0.019	0.002
		pT4	1	9.255	6.217	0.054	0.004
	Lymph nodes invasion	pN0	30	1.376	0.911	0.014	0.009
		pN1 & pN2	12	1.488	0.645	0.011	0.003
AJCC		AJCC I	21	1.681	0.784	0.020	1.681
		AJCC II	15	1.376	1.061	0.008	1.376
		AJCC III	6	1.295	1.082	0.019	1.295
Pack-years (PYs)		≤30 PYs	11	1.000	0.524	0.023	0.004
		31-45 PYs	13	2.197	1.952	0.036	0.008
		>45 PYs	17	1.132	0.760	0.006	0.006
Type of the surgery		Lobectomy	33	1.287	0.836	0.013	0.007
		Pneumonectomy	9	2.925	1.535	0.014	0.003
		Bilobectomy	4	0.466	1.252	0.009	0.004
		Segmentectomy	1	22.100	16.898	0.052	0.010

*NLNT, normal-looking neighboring tissue; pTNM, International System of Clinico-Morphological Classification of Tumors (TNM, Tumor Node Metastasis) according to the WHO Histological Typing of Lung Tumors; AJCC, American Joint Committee on Cancer Staging (AJCC staging) according to the IASCLC Staging Project 7th edn*.

The validation of expressional data with the Genomic Data Commons also revealed significantly decreased *TIMP3* level in cancer tissues in comparison to controls in both LUAD (*p* < 0.001; UMW) and LUSC projects (*p* < 0.001; UMW). Whereas, *MMP2* was expressed on a comparable level in both tissues in LUAD and LUSC projects. *TIMP3* expression was higher in the AC subtype in comparison to SCC (34–36), see Table 3B. In the present study, we have also compared the *TIMP3/MMP2* expression ratios in NLNT vs. healthy lung tissues. The experimentally-assessed expression of *TIMP3* in NLNT was ~109 times lower than the *MMP2*. In the RNA-seq normal tissues datasets (gtexportal.org, proteinatlas.org: controls for LUSC and LUAD projects) *TIMP3* expression was 3.35–7.77 higher than *MMP2* (see Supplementary Figure 4).

### microRNA Expression

MiR expression was assessed among 43 patients (see Table 5). Both miRs were expressed at comparable levels before and after surgery (*p* = 0.681 and *p* = 0.334, respectively, Wilcoxon test). The relative distribution and symmetry of miRs expression within the analyzed groups (before vs. after surgery; AC vs. SCC subtype) are presented in section Materials and Methods in Supplementary Materials and Supplementary Figure 3). The type of surgery did not affect the expression of postoperative miRs (*p* = 0.202 for *miR-17* and *p* = 0.202 for miR-20a, Kruskal-Wallis test). No statistically significant observations were made for miR levels in terms of age, gender, or TNM tumor classification. Besides, no significant correlations were found between miRs and tobacco intake (measured in pack-years), neither before nor after tumor resection. There were observed positive correlations between the miR expression before and after surgery (see Figure 1).

**Table 5:** Clinical and pathological features: median expression level (RQ value) of tested miRs.

**Clinical and pathological features**			***N***	***miR-17* before surgery**	***miR-17* after surgery**	***miR-20a* before surgery**	***miR-20a* after surgery**
Entire group			43	0.459 (IQR: 0.088–1.118)	0.667 (IQR: 0.135–1.102)	2.181 (IQR: 0.896–13.134)	1.499 (IQR: 0.907–7.410)
Gender		Women	17	0.496	0.876	2.017	1.141
		Men	26	0.408	0.542	2.489	1.893
Age group		≤60	8	0.081	0.295	4.919	4.856
		>60	35	0.496	0.868	2.017	1.152
Histopathological type		AC	21	0.348	0.593	2.871	1.687
		SCC	21	0.773	0.876	1.294	1.141
pTNM	Tumor size	pT1	10	0.533	0.596	2.359	1.775
		pT2	25	0.396	0.667	2.523	1.499
		pT3	6	0.904	0.634	1.202	1.848
		pT4	1	0.500	0.587	9.516	7.619
	Lymph node invasion	pN0	30	0.377	0.701	2.660	1.429
		pN1 & pN2	12	0.638	0.380	1.644	2.632
AJCC		AJCC I	20	0.020	0.006	0.427	0.701
		AJCC II	17	0.008	0.011	0.358	0.627
		AJCC III	5	0.019	0.003	1.160	0.391
Pack-years (PYs)		≤30 PYs	12	0.338	0.596	3.384	1.775
		31–45 PYs	15	0.776	0.895	1.289	1.117
		>45 PYs	15	0.292	0.137	3.424	7.300
Type of the surgery		Lobectomy	33	0.353	0.525	2.834	1.939
		Pneumonectomy	9	0.776	0.934	1.289	1.071
		Bilobectomy	4	0.459	0.348	2.181	2.877
		Segmentectomy	1	0.042	0.667	23.800	1.499

*pTNM, International System of Clinico-Morphological Classification of Tumors (TNM, Tumor Node Metastasis) according to the WHO Histological Typing of Lung Tumors; AJCC, American Joint Committee on Cancer Staging (AJCC staging) according to the IASCLC Staging Project 7th edn*.

**Figure 2:**
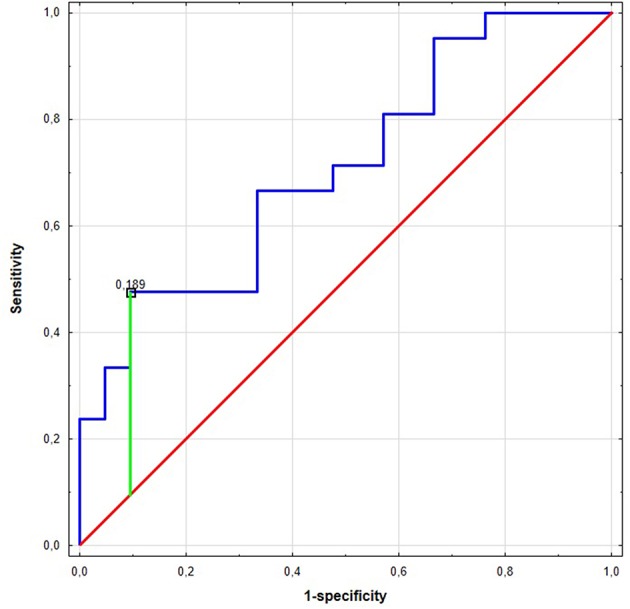
The Receiver Operating Characteristic (ROC) curve of *miR-17* in the NCSLC subtype classification. The best cut-off point for AC detection was equal to ≤0.189 for *miR-17* (Youden's J statistic). The area under the curve (AUC) was equal to AUC 0.710 (95% CI: 0.554–0.865), its predictive value was as follows: specificity−90%, positive predictive value (PPV)−83%, sensitivity−48%, and negative predictive value (NPV)−63%.

Significant differences were found for preoperative miR expression among NSCLC subtypes (see Supplementary Figure 3). The preoperative *miR-17* expression was significantly higher among SCC patients compared to AC (*p* = 0.02, UMW test). For preoperative *miR-20a*, the opposite dependence was observed (*p* = 0.02, UMW test). The presurgical *miR-17* and *miR-20a* expression levels were used to establish the NSCLC subtype determination test. AUC for *miR-17* classifier was 0.710 (95% CI: 0.554–0.865). For the optimal cut-off point (≤0.189; Youden's J statistic), the specificity and positive predictive values (PPV) in detecting AC were equal to 90 and 83%; sensitivity and negative predictive value (NPV) were equal to 48 and 63%, respectively (see Figure 2). Moreover, presurgical *miR-20a* expression may be used to distinguish the NSCLC subtypes with AUC of 0.71 (95% CI: 0.554–0.865). For the optimal cut-off point (≥5.295 for AC; Youden's J statistic) the specificity and PPV in detecting AC were equal to 90 and 83%; sensitivity and NPV were 48 and 63%, respectively.

The validation of the miR expressional data with the Genomic Data Commons (34–36) revealed significantly decreased *miR-17* expression in controls, compared to cancer group in both LUSC and LUAD projects. The miR-20a expression in cancer vs. control groups was opposite in LUSC and LUAD projects (see Table 3B).

### Correlation of Gene and microRNA Expression

The analysis of miR correlation with gene expression was performed on 39 patients with overlapping data. No correlations were found in cancer tissue for any of the samples. Weak correlations were discovered between *TIMP3* expression in the NLNT and postoperative miRs: a positive correlation for *miR-17* (*R* = −0.334, *p* = 0.037; r_s_) and a negative correlation for *miR-20a* (*R* = 0.330, *p* = 0.04; r_s_). Similarly in SCC subtype the *TIMP3* expression in NLNT correlated positively with *miR-17* (before: *R* = 0.474, *p* = 0.03; after: *R* = 0.549, *p* = 0.01; r_s_), and negatively with *miR-20a* (before: *R* = −0.474, *p* = 0.03; after: *R* = −0.549, *p* = 0.01; r_s_) (see Figure 1). No statistically significant correlations between miR and gene expression were observed within the AC group.

## Discussion

The high mortality and late detection rates associated with NSCLC create an urgent need for developing new biomarkers enabling early detection. Hence, it is essential to identify candidate biomarkers among genes and microRNAs regulating the ECM remodeling present in early stages of NSCLC, which can help distinguish subtypes. Our present study aimed to evaluate the relative expression of selected genes and miRs engaged in ECM remodeling in histopathologically-confirmed NSCLC, as candidates for molecular biomarkers.

### Decreased *TIMP3* Expression in Normal-Looking Neighboring Tissue

Our analysis found *TIMP3* expression to be significantly lower in cancer tissue than in normal looking neighboring tissue. A similar tendency was also observed in the analysis of data from the Genomic Data Commons, where *TIMP3* was significantly decreased in cancer tissues in comparison to controls in both analyzed AC and SCC cohorts (34, 35). Under physiological conditions, any increase of metallopeptidase expression and its activity would be controlled by specific non-covalent binding of the TIMP3 protein, resulting in MMP inhibition (17). In our study, we have noted the positive correlation between *TIMP3* and *MMP2* expression in NLNT, confirmed in the AC subtype, but not in SCC. On the other hand, the observed in the present study, silencing of the metalloproteinase inhibitor *TIMP3* suggests that the proteins taking part in ECM remodeling can display intensified activity. Such remodeling, especially un-controlled ECM proteolysis, may result in more significant cancer cell proliferation and migration. It was previously demonstrated that restoring *TIMP3* function by blocking the expression of its suppressor gene *EZH2* (using RNA interference) led to subsequent inhibition of cancer cell migration (39). Those findings are not entirely concordant with those of Kumaki et al. who report that increased immunoexpression of different metalloproteinase inhibitors correlated with tumor aggressiveness. The TIMP2 protein was significantly stronger in the invasive areas than the lepidic areas of Invasive Pulmonary Adenocarcinoma (11); this has been attributed to increased TIMP2 expression causing elevated ECM accumulation in the invasive tumor cells, resulting in fibrous scar formation (11).

Cigarette smoking is overwhelmingly tied with SCC rather than AC (40). Regarding smoking status in NSCLC subtypes, we have observed significantly lower *TIMP3* expression in NLNT in long term smokers in SCC subtype, but not in AC. Smoking-induced *TIMP3* downregulation may be one of the molecular causes of cancerogenesis among SCC patients. It was earlier demonstrated that molecular changes like microsatellite instability (MSI) or suppressor gene hypermethylation could occur in histologically-normal epithelia or macroscopically-unchanged tissue adjacent to the resected tumors in smoking patients with primary lung cancer. Also, in smokers without cancer, the loss of heterozygosity and MSI were detected in histologically-normal distal bronchial epithelium (41–44).

### *MMP2* Expressed on Comparable Level in Both Tissues

MMPs are typically expressed at moderate levels; their expression rapidly grows in response to tissue injury (8, 14) and the course of inflammation processes or cancer development. It was previously reported that MMP2 immunoexpression was significantly higher in the lung cancer group than among the healthy control group (45). On the other hand, ~50% of the NSCLC patients revealed stronger upregulation of the *MMP2* in the fibroblasts neighboring the lesion, than in the tumor itself (12). MMP2 protein may act as an extracellular matrix modulator in fibroblast cells, enabling malignant transformation by ECM degradation and the creation of a suitable microenvironment for vessel growth (12). In our study, performed on paired tissues from NSCLC patients, the *MMP2* expression was on comparable level in cancer tissue and NLNT. The lack of the *MMP2* expression differences among tissues, together with the observed *TIMP3/MMP2* ratio distortion in NLNT (strongly decreased *TIMP3* expression in comparison to *MMP2*, compared to the data from RNAseq studies), indicate that ECM remodeling may be observed some distance from the lesion center. Previous studies have linked the molecular changes in surrounding tissue, mediated by miRs, to the field cancerization effect (43). The phenomenon of tumor promoting (oncogenic) activity of the *MMP2* was previously demonstrated in colorectal cancer, where elevated *MMP2* mRNA levels in “healthy” tissue surrounding the lesion were significantly higher in patients with metastatic cancer than in non-metastatic lesions (46).

### miR Expression From Serum Exosomes—Significantly Differs Between NSCLC Subtypes

Many studies report that both *miR-17* and *miR-20a* influence tumor formation and cancer progression in various organs and tissues throughout the body (47–50); however, the vast majority of such papers overwhelmingly describe experiments performed using various cancer cell lines. The present study assesses their expression in exosomes from peripheral blood samples of NSCLC patients, collected before and after surgical removal of the tumor, and compares our findings with data retrieved from TCGA. This approach to miR analysis is described in a few publications (51, 52). In the present study, the *miR-20a* expression levels in pre- and postsurgical samples correlated positively. The miRs produced by the tumor to control the process of matrix remodeling can be observed in patients serum some days after cancer removal. On the other hand Zhang et al. demonstrated that after surgery the lower expression of *miR-20a* and four other miRs after surgery might indicate the *miR-20a* originates from tumor tissue (52). While comparing *miR-17* expression before and after surgical treatment, no significant difference was found. Nevertheless, our study is the first to examine the change in *miR-17* expression among NSCLC patients before and after surgical treatment.

Our research indicates that the miR expression pattern in serum obtained before surgery significantly differs between NSCLC subtypes. *MiR-17* expression was higher among patients with SCC than those with AC, which is concordant with Molina-Pinelo et al. study (53) and was also confirmed by TCGA data (34, 35). Analysis of this dependency was used to create a classifier differentiating between NSCLC subtypes (Figure 2). Strongly decreased expression of *miR-17* can be treated as a hallmark of AC subtype with 90% specificity, where a mean RQ value over 0.189 FC can indicate a SCC subtype with 48% specificity. The opposite trend was observed for *miR-20a*: its expression was significantly increased in AC in comparison to the SCC group.

Our results are in accordance with the analysis of TCGA data, which revealed a higher number of *miR-20a* transcripts among AC patients than those with SCC. *MiR-20a* has previously been described as SCC subtype biomarker; however, these studies compared samples from SCC patients with those of healthy volunteers, or with postoperative samples from the same patient (only SCC subtype). Zhang et al. propose a panel of three microRNAs (including *miR-20a*) as a potential diagnostic marker for distinguishing male lung SCC patients from healthy volunteers (54). Aushev et al. propose a panel of five miRs (*miR-205*,−*19a*,−*19b*,−*30b*, and *-20a*) as a SCC biomarker; these five miRs were found to be upregulated in exosomes extracted from the peripheral blood of SCC patients before surgery compared to postoperative samples (55). However, neither study performed any assessment regarding other NSCLC subtypes.

As no literature comparing *miR-20a* expression among AC vs. SCC patients could be found, our study appears to be the first to propose *miR-20a* as biomarker for distinguishing NSCLC subtypes, following an analysis of miR expression in SCC and AC. This can be essential in case of advanced lung cancer (confirmed with low-invasive methods like bronchoscopy or lung needle biopsy) in AJCC clinical stage IIIA or IIIB, where chemotherapy can be used as induction treatment before surgery or as a complementary treatment for radiotherapy (3–5). Preoperative subtype diagnosis may help to select better first-line chemotherapy schemes in advanced NSCLC. Different outcomes were observed for squamous vs. non-squamous cell carcinomas: i.e., a better response was observed for pemetrexed treatment in non-squamous NSCLCs compared to SCC (56). As no correlations of miR expression with tumor grading and staging (TNM and AJCC) were found, those miRs probably would not be useful as preoperative circulating biomarkers of cancer stage.

### The Correlations of Gene and miR Expression

**Figure 3:**
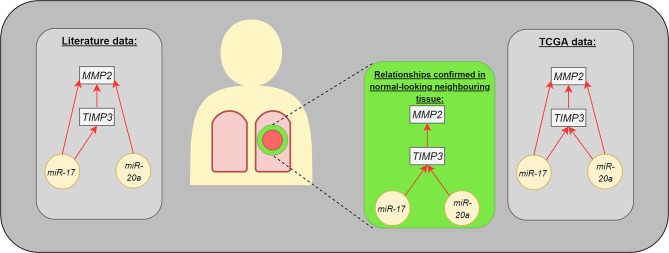
The interaction between analyzed molecules (genes and microRNAs). The gray boxes present the dependencies published in medical literature (left) and TCGA data (right). The middle part presents the statistically significant differences observed in our study: microRNAs were assessed in postoperative blood sample, genes in normal-looking neighboring tissue from surgical margin.

The present study identified correlations regarding gene expression (*TIMP3* vs. *MMP2*) or genes and miRs (*TIMP3* vs. *miR-17* and *miR-20a*) in the normal-looking neighboring tissue, which were not detected in the center of the cancer lesion (Figure 3). Such changes in miR expression can be prompted by more complex regulation of ECM remodeling in the cancer neighborhood or could be characteristic of NSCLC subtype. Moreover, our results are in concordance with miR expressional data from the Genomic Data Commons, where the studied miRs presented different trends among subtypes: *miR-20a* was upregulated among tested LUAD patients, but not LUSC.

Due to the gene location (13q31) the analyzed miRs were observed to be expressed jointly as a 2 gene cluster or act as a part of larger *miR-17-92* cluster which has been overexpressed in LC, colorectal cancer, and hepatocellular carcinoma (30, 57). We did not observe any joint *miR-17/miR-20a* expression pattern: the preoperatively *miR-17* expression was higher in SCC, while *miR-20a* was higher in AC, and miRs correlated in opposite way with *TIMP3* in NLNT.

In physiological conditions, the *TIMP3* expression would be triggered by the ECM remodeling signals to counterpart the MMP activity (17). *TIMP3* silencing is associated with the intensified ECM remodeling and cancer cell migration (39). Such ECM remodeling in the tumor surrounding tissue can be forced by the rapid growth of the lesion and its rigidity: when the diameter of the tumor increases, it exerts pressure on neighboring tissues and blood vessels (48). Surprisingly we have observed the positive correlation of *TIMP3* in NLNT with *miR-17* expression (in the entire study cohort and SCC subtype, not detected in AC) as the increase of the *miR17* is linked to the angiogenesis, though we hypothesize that *TIMP3* expression may be activated by indirect miR action. On the other hand, we have observed a significant increase of the preoperative *miR17* expression in SCC compared to AC, which can partially explain the observed positive correlation with *TIMP3* decrease.

The observed in our study negative correlation of *TIMP3* with *miR-20a* expression (both preoperative and postoperative *miR20a* in SCC subtype, post *miR20a* in the entire study cohort), can be explained as epigenetic silencing of the genes controlling the ECM remodeling. The *miR-20a* action has been previously linked to the induction of vascular changes in invasive breast carcinomas (48) and metastasis in gastric cancer (50). *TIMP3* silencing mediated by *miR-20a* may be treated as a hallmark of substantial ECM deregulation already present in the NLNT. Remodeling encourages further growth of the lesion and induces local hypoxia, which in turn can encourage the development of new blood vessels growth (48, 57). The observed strong downregulation of *TIMP3* and disturbed *MMP2/TIMP3* expression ratio can be explained as crucial activities in the ECM remodeling process, enabling creation of a microenvironment conducive to tumor growth (57).

## Conclusions

- The *MMP2* expression on comparable level in both tissues, together with strong *MMP2* vs. *TIMP3* upregulation in NLNT (compared to the healthy controls data from HPA RNA-seq project or Genomic Data Commons), indicate the metalloproteinase mediated ECM remodeling can occur in the distance from the center of the lesion.
- The *TIMP3* silencing observed in the normal-looking neighboring tissue and its negative correlation with presurgical *miR-20a* expression from serum exosomes (in SCC subtype) suggest the role of miRs in ECM remodeling
- miR expression pattern in serum obtained before surgery significantly differs between NSCLC subtypes. Preoperative serum miR examination can be considered as a useful biomarker for the neoadjuvant therapy planning for patients with confirmed lung cancer and clinical AJCC stage III A/III B. Furthermore, the proposed miR-based classifier does not require the use of minimally invasive diagnostic methods, such as biopsy or broncho-alveolar lavage, just a simple extraction of miR from serum exosomes.
- The downregulation of *TIMP3* in long-term smokers and the decrease of presurgical *miR-17* expression, can be regarded as potential SCC subtype markers.

## Strengths and weaknesses of the study

The strengths of this study are:

- the prospective design;
- inclusion of the NSCLC patients that were not treated with potentially mutagenic chemotherapy or radiotherapy before the surgery;
- analysis in the most common NSCLC subtypes: AC and SCC;
- analysis of *miR-17* and *miR-20a* expression before and after surgical treatment;
- analysis of *MMP2* and *TIMP3* expression in both cancer lesion and the normal-looking neighboring tissue (from surgical margin);
- validation of the obtained results with the available sequencing datasets from HPA RNA-seq project, or Genomic Data Commons.

The weaknesses of the study are:

- a relatively low number of studied patients, despite a proper distribution among NSCLC subtypes.
- Difficulty confirming the cancer origin of the analyzed miRs—miRs were extracted from exosomes circulating in blood.
- Expression analysis performed only at the mRNA level, not assessed on the protein level. IHC analysis was not provided either in our study nor in the TCGA validation dataset.

## Data Availability Statement

The datasets generated for this study are available on request to the corresponding author.

## Ethics Statement

The study was performed in accordance with the Helsinki Declaration and the ethical proceedings approved by the Ethical Committee of the Medical University of Lodz, Poland, no. RNN/140/10/KE. The patients/participants provided their written informed consent to participate in this study.

## Author Contributions

KC created the concept of the study and provided the final version. KC and DP-L design the study. KC, BS, MB, and MK performed laboratory procedures, conducted statistical analysis, and wrote the manuscript. JK, AA, DP-L, and EB-L revised the manuscript.

### Conflict of Interest

The authors declare that the research was conducted in the absence of any commercial or financial relationships that could be construed as a potential conflict of interest.

## Supplementary Material

Supplementary Figure 1 Diagram of the interactions between genes selected for the study (*TIMP3, MMP2*) and miRs targeting them. MiRs selected using the databases microRNA.org and mirtarbase.mbc.nctu.edu.tw.

Supplementary Figure 2 Violin plot describing the distribution of *MMP2* and *TIMP3* gene expression in tumor and normal-looking neighboring tissue **(A,B)**, as well as in NSCLC subtypes **(C,D)**. The violin plot analysis represents the kernel density plot of the analysis of binary classified data. SCC, Squamous Cell Carcinoma; AC, Adenocarcinoma.

Supplementary Figure 3 Violin plot describing distribution of *miR-17* and *miR-20a* expression in tumor and normal-looking neighboring tissue: **(A,B)** and in NSCLC subtypes **(C,D)**. The violin plot analysis represents the kernel density plot in the analysis of binary classified data. SCC, Squamous Cell Carcinoma; AC, Adenocarcinoma.

Supplementary Figure 4 Box-and-whisker plot representing the expression of *TIMP3* in tumor and normal-looking neighboring tissue from surgical margin (*p* = 0.01; Wilcoxon test).

Supplementary Table 1 Expression level of analyzed genes in control tissue from RNA-seq analysis and normal-looking neighboring tissue.
