# Supplementary material for: A Strong Decrease in TIMP3 Expression Mediated by the Presence of miR-17 and 20a Enables Extracellular Matrix Remodeling in the NSCLC Lesion Surroundings
Source: Front Oncol. 2019 Dec 13;9:1372. doi: 10.3389/fonc.2019.01372 (PMC6923190; doi:10.3389/fonc.2019.01372)
Supplement: Supplementary Figure 1 — Diagram of the interactions between genes selected for the study (TIMP3, MMP2) and miRs targeting them. MiRs selected using the databases microRNA.org and mirtarbase.mbc.nctu.edu.tw. [file Table_1.DOCX]

Supplementary Material

1. **Selection of microRNA molecules**

MicroRNAs targeting *MMP2* and/or *TIMP3* in LC were chosen based on the TCGA datasets, containing RNAseq results of NSCLC patients with AC (LUAD project) and SCC (LUSC project). Two datasets for AC and SCC, each containing cancer group and a control group, were downloaded using the TCGA biolinks R package.

Further validation, using data retrieved from public microRNA databases (microRNA.org; mirtarbase.mbc.nctu.edu.tw), indicated that *miR-20a* silences *MMP2* expression and *miR-17* targets both *MMP2* and *TIMP3* (see Supp.Fig. 1). In the performed literature search (PubMed query on miR & ECM remodelling & cancer) many studies indicated that both miRs have a significant impact on the development of cancer throughout the body [31–38].


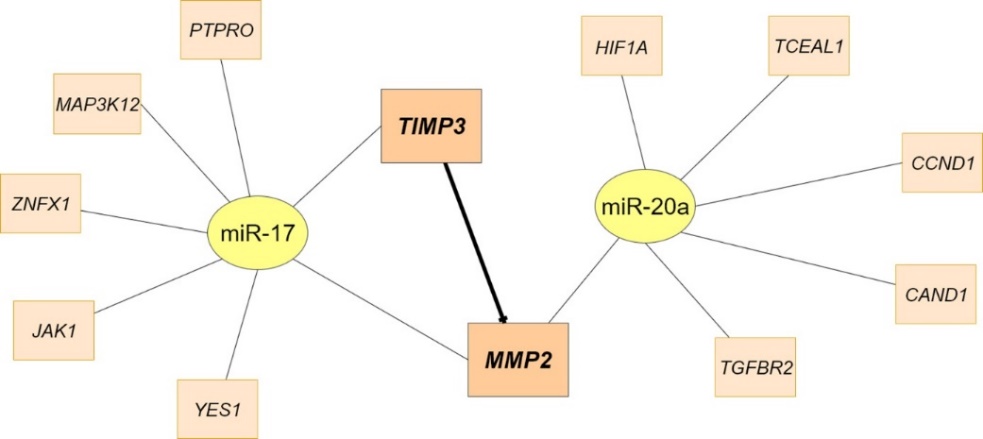


**Supplementary** **Figure 1.** Diagram of the interactions between genes selected for the study (*TIMP3, MMP2*) and miRs targeting them. MiRs selected using the databases microRNA.org and mirtarbase.mbc.nctu.edu.tw.

1. **Violin plots presenting the distribution of genes and miR expression**

Violin plots represent the probability density function of a random variable estimated with the kernel density estimation (KDE) analysis for non-parametric data. Violin plots allow to compare two different distributions of gene and miR expressions among the examined groups. The violin plot representation appears non-symmetrical regarding tissue type for the expression of *TIMP3*, but not *MMP2* (**Supp. Fig.2**). Also, the violin plot representation of the relative miR expression reveals a lack of symmetry for *miR-17* distribution (**Supp. Fig.3**). Lack of symmetry is associated with differences in gene and miR expression, and thus distribution, in the compared groups. The observed lack of symmetry in the case of *TIMP3* corresponds to a statistically significant difference in expression in cancer tissue *vs* NLNT (Supp. Fig 4).


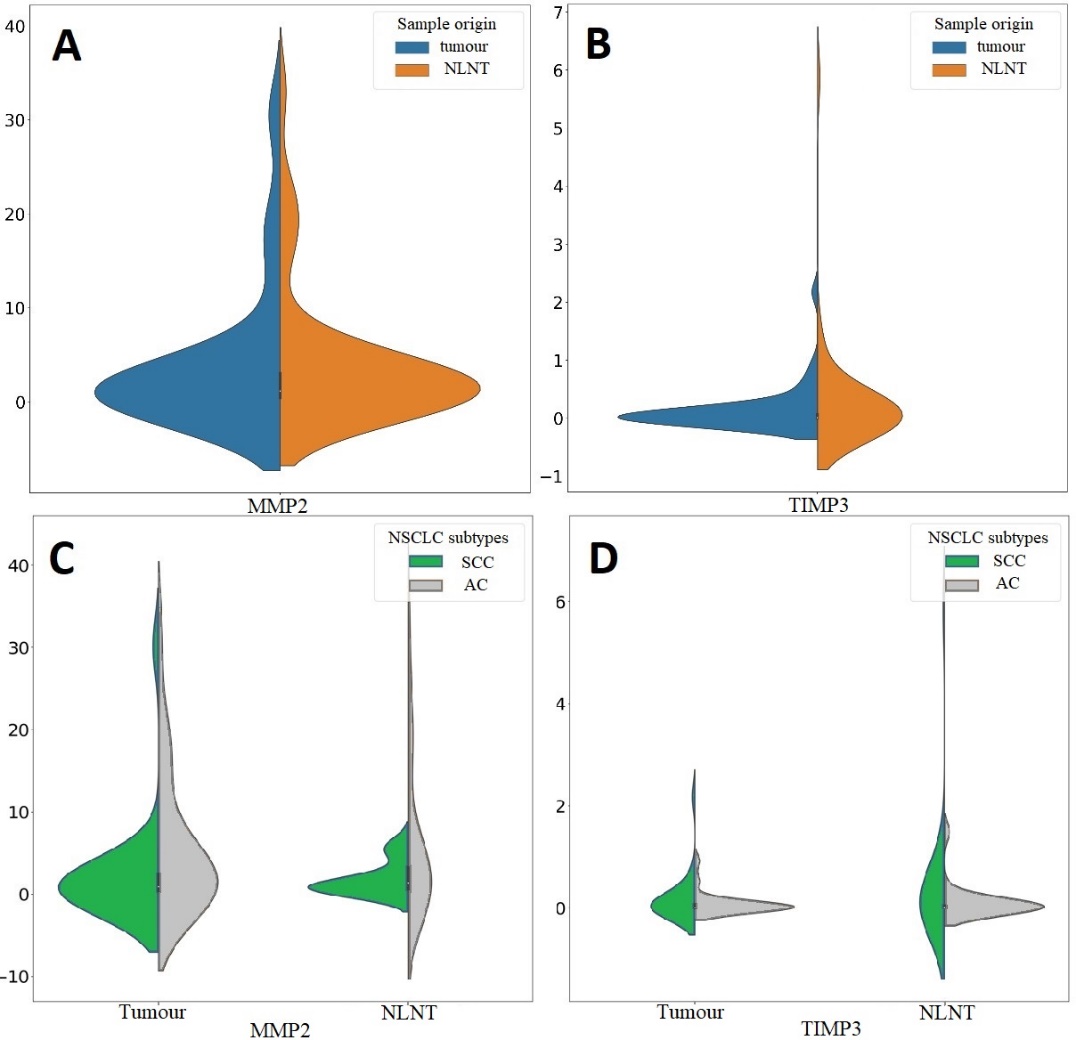


**Supplementary Figure 2.** Violin plot describing the distribution of *MMP2* and *TIMP3* expression in tumour and NLNT (2.A & 2.B), as well as in NSCLC subtypes (2.C & 2.D). **Legend**: **NLNT** **-** normal-looking neighbouring tissue; **SCC** - Squamous Cell Carcinoma; **AC** - Adenocarcinoma.
Additional information: The presented gene expression is compared to calibrator (Human Lung Total RNA). **(A)** *MMP2* was downregulated in tumor tissue and upregulated in NLNT in comparison to calibrator (median value: 0.900 vs .422); **(B)** *TIMP3* was downregulated in both cancer and NLNT (MVs: 0.013 vs. 0.006); **(C)** *MMP2* expression was elevated in AC compared to SCC both in cancer (MVs: 1.252 vs. 0.530) and NLNT (MVs: 1.575 vs. 1.015); **(D)** *TIMP3* expression were similar in AC and SCC in cancer (MVs: 0.006 vs. 0.004) and NLNT (MVs: 0.013 vs. 0.013) subtypes.


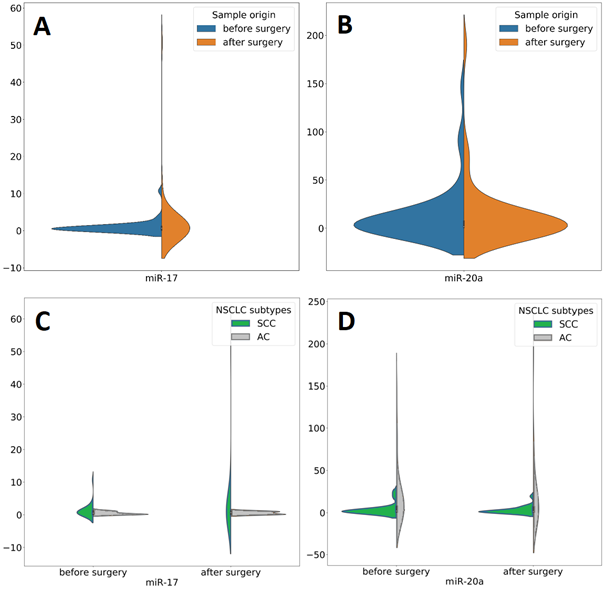


**Supplementary Figure 3.** Violin plot describing distribution of *miR-17* and *miR-20a* expression in tumour and normal-looking neighbouring tissue: (3.A & 3.B) and in NSCLC subtypes (3.C & 3.D). **Legend**: **SCC** - Squamous Cell Carcinoma; **AC** - Adenocarcinoma.

Additional information: **(A)** *miR-17* expression is increased after surgery (MVs: 0.459 vs. 0.667)
**(B)** *miR-20a* expression *is* decreased after surgery (MVs: 2.181 vs. 1.499); **(C)** The preoperative miR-17 expression was higher among patients with SCC vs. AC (MVs: 0.773 vs. 0.348); **(D)** The preoperative *miR-20a* expression was higher among patients AC vs. SCC (MVs: 2.871 vs. 1.294).

1. **The *TIMP3* expression in tumour tissue and NLNT**


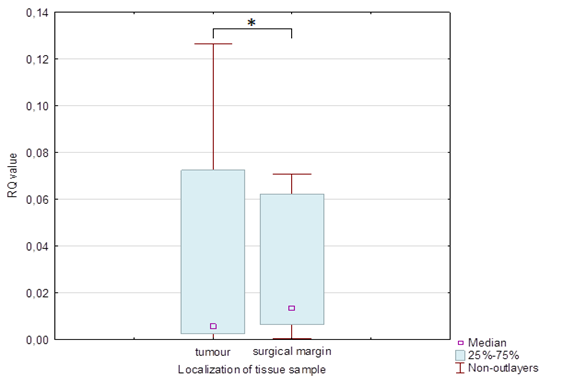


**Supplementary Figure 4.** Box-and-whisker plot representing the *TIMP3* expression in cancer tissue and NLNT from the surgical margin (p=0.01; Wilcoxon test).

1. ***TIMP3/ MMP2* expression ratio in NLNT vs healthy tissues**

We have compared the *TIMP3/ MMP2* gene expression ratio in healthy tissues RNA-seq data with the experimentally-assessed expression of genes in NLNT obtained in our study (**Sup. Tab. 1)**.

| **Tissue** | ***TIMP3* expression** | ***MMP2* expression** | ***TIMP3/MMP2* ratio** | **Source of data** |
| --- | --- | --- | --- | --- |
| Healthy lung tissue | 234.24 RPKM (1) | 62.96 RPKM (2) | 3.77 | HPA RNA-seq normal tissues project |
| Healthy lung tissue | 487.2 RPKM (3) | 145.3 RPKM (4) | 3.35 | The Genotype –Tissue Expression |
| Controls for SCC project | 33445.33 FPKM | 7945.22 FPKM | 4.21 | Genomic Data Commons [39] |
| Controls for AC project | 38223.79 FPKM | 4919.22 FPKM | 7.77 | Genomic Data Commons [40] |
| **Normal-looking neighbouring tissue** | **0.013 FC** | **1.422 FC** | **0.009** | **Current study** |

**Supplementary Table 1. Expression level of analysed genes in control tissue from RNA-seq analysis and normal-looking neighbouring tissue.** Data retrieved from HPA RNA-seq normal tissues project at Genomic Data Commons (gdc.cancer.gov). **Legend**: **RPKM** – Reads Per Kilobase Million; **FPKM** – Fragments Per Kilobase Million; **FC** – Fold Change.

Presented data were obtained from: (1) https://gtexportal.org/home/gene/TIMP3; (2) https://gtexportal.org/home/gene/MMP2; (3) https://www.proteinatlas.org/ENSG00000100234-TIMP3/tissue/lung; (4) https://www.proteinatlas.org/ENSG00000087245-MMP2/tissue/lung.

In our present study, the experimentally-assessed expression of *TIMP3* was ~109 times lower than that of *MMP2* (**Sup. Tab. 1).** According to data retrieved from the HPA RNA-seq normal tissues project (gtexportal.org) and The Genotype-Tissue Expression (proteinatlas.org) the transcription level of *TIMP3* in healthy lung tissue was ~3.35-3.77 times higher than *MMP2*. In controls for LUAD and LUSC TCGA projects, the *TIMP3* is ~4.21 – 7.77 higher than *MMP2* [39,40].
